# Impact of Multiplayer Online Role-Playing Games upon the Psychosocial Well-Being of Adolescents and Young Adults: Reviewing the Evidence

**DOI:** 10.1155/2013/464685

**Published:** 2013-03-28

**Authors:** Jonathan Scott, Alison P. Porter-Armstrong

**Affiliations:** ^1^The Institute of Nursing and Health Research, Faculty of Life and Health Sciences, University of Ulster, Newtownabbey BT37 0QB, UK; ^2^Rehabilitation Sciences, The Institute of Nursing and Health Research, Faculty of Life and Health Sciences, University of Ulster, Newtownabbey BT37 0QB, UK

## Abstract

*Introduction*. For many people, the online environment has become a significant arena for everyday living, and researchers are beginning to explore the multifaceted nature of human interaction with the Internet. The burgeoning global popularity and distinct design features of massively multiplayer online role-playing games (MMORPGs) have received particular attention, and discourses about the phenomenon suggest both positive and negative impact upon gamer health. *Aim*. The purpose of this paper was to critically appraise the research literature to determine if playing MMORPGs impacts upon the psychosocial well-being of adolescents and young adults. *Method*. Initial searches were conducted on nine databases spanning the years 2002 to 2012 using key words, such as online gaming, internet gaming, psychosocial, and well-being, which, in addition to hand searching, identified six studies meeting the inclusion and exclusion criteria for this review. *Results*. All six studies strongly associated MMORPG playing with helpful and harmful impact to the psychosocial well-being of the populations under study; however due to the methodologies employed, only tentative conclusions may be drawn. *Conclusion*. Since both helpful and harmful effects were reported, further multidisciplinary research is recommended to specifically explore the clinical implications and therapeutic potentialities of this modern, growing phenomenon.

## 1. Introduction

There can be little doubt that the use of the Internet has become a significant aspect of modern living, bringing benefits to users in terms of access to information and flexibility of communication. Even so, certain aspects of Internet use are beginning to come under increasing scrutiny. Recently, the term *Problematic Internet Use* has been used to describe a syndrome of cognitive and behavioural symptoms that result in a wide range of negative consequences, including physical harm and psychosocial adversities [1–3]. Indeed there is a body of opinion suggesting that the term *internet addiction* should be included in the forthcoming fifth edition of the diagnostic and statistical manual of mental disorders [4]. However, research suggests that individuals do not develop problems with the Internet in and of itself, but rather with the various activities it enables [5–7]. 

Despite the increasing pervasiveness and variety of online activities in adolescent's lives, little is known about any associated short- or long-term health implications of use [8]. Of particular interest are those activities which involve online community interaction, and questions have been raised about the ability of these applications to influence behaviour and induce or support pathological thinking [9]. Online gaming is one such activity and is the latest iteration of the well-established leisure occupation of video and computer gaming. It has become a significant global phenomenon, with one source estimating that there are more than 217 million online gamers worldwide [10] and other statistics estimating that one in four internet users access sites that offer gaming [11]. Indeed, market research indicates that the average number of hours spent each week on online gaming is increasing, with 12–14 year olds spending the most time on these games [12].

An online game is a digital game that utilises a live network connection in order to be played and is usually done through a games console, a portable gaming device, or a personal computer [13]. As well as the traditional offline gaming experience of “continuous scoring, promotion, immediate feedback, and achievement of self-satisfaction” [14], which research has shown can have many adverse health consequences [15, 16], online gaming allows for social interaction amongst gamers in a shared virtual space [17] and consequently may be more problematic for some individuals [18].

The most popular genre or type of online game that of massively multiplayer online role-playing games (MMORPGs) and the small amount of research exploring online gaming has tended to focus on this genre. MMORPGs allow gamers to create their own avatar to explore and play with others from across the globe in self-contained, persistent, and immersive online worlds. By design, these games run in real time are highly social and competitive in nature, and call for a high level of commitment and cooperation amongst game users [19]. Consequently there are growing concerns that the significant requirements of playing MMORPGs may facilitate compulsive or addictive use. It has been reported that in order to create more time for computer games, players will neglect sleep, diet, hobbies, exercise, and socialising [20] and that there is evidence to associate poor decision making [21], depressive symptoms, and suicidal ideation with excessive digital gaming [22]. However, it should be noted that studies have also reported that users are deriving a great deal of satisfaction and benefit from engaging in these games [23–26]. Clearly a heterogeneous clinical picture is beginning to emerge of how online gaming is impacting the psychosocial well-being of gamers. For the purposes of this review “psychosocial well-being” is understood as an “array of constructs which reflect the quality of intrapersonal and interpersonal functioning” [27]. Moreover, since playing MMORPGs is becoming an increasingly significant occupation of leisure for many adolescents and young adults, the aim of this critical review is to examine the published literature and to critically appraise the evidence to determine the impact, if any, of playing MMORPGs upon the psychosocial well-being of adolescents, emerging adults, and young adults.

## 2. Materials and Methods

### 2.1. Search Strategy

A systematic search of the period January 2002 to January 2012 was conducted on nine databases (AMED, ASSIA, Cochrane Database of Systematic Reviews, CINAHL, Embase, MEDLINE, OTDatabase, ProQuest, and PsycINFO) in order to identify relevant literature. The term online gaming along with the alternatives internet gaming, computer gaming, and MMORPG were searched concurrently with mental health along with the alternatives psychosocial, well-being, and health status. All primary research study designs in the English language were considered; however, studies were required to have a number of further features in order to be included in the review. First of all, the study had to clearly indicate that MMORPG playing was the subject of the investigation/exploration. It was very common to read articles that looked at “gaming” in general, but from which it was not possible to identify if MMORPG playing was an element of the study. Secondly, studies needed to focus on one or more specific aspects of psychosocial well-being by including a psychosocial outcome measure. Finally, for inclusion in the review, it was necessary for studies to explicitly specify any of the following demographic groupings of “adolescent,” “emerging adult,” or “young adult” as the population under study. In the following review, these groupings are together taken to be encompassed by the age range of 10–30 years old. Again it was very common to read articles that included vast age ranges in participants, and it was impossible to isolate particular groups from the data.

 As might be expected, in line with the emerging nature of this area of study, researchers have engaged with this phenomenon by using a variety of different terms when describing online gaming such as MMORPG playing; however, very few studies carry clear, explicit definitions and descriptions of both the gaming and the gamers under study [18]. Furthermore, it would seem that difficulties exist in the classification of this phenomenon in the literature, which has provided little agreement as to how online gaming should be conceptualised. Product refinement and technological progression constantly push the barriers of gaming experience and possibilities; yet, studies exploring this new technology are still to be found in categories such as “video games” and “computer games.” Consequently, initial searches identified four hundred and sixty seven articles and of those, only thirteen were selected as being potentially relevant for this review. The removal of duplicates reduced this number to eleven, and the application of inclusion/exclusion criteria further reduced this number to two. Four additional articles were identified through hand searching reference lists, and as a result, a total of six articles were reviewed.

### 2.2. Critical Appraisal

The study of the psychosocial *components *of online gaming has a small but growing evidence base in the recent literature; however, studies investigating the psychosocial *impact *of this phenomenon are scarce. In terms of negative health associations, a number of studies claim to have identified a significant number of “addicted” online gamers with data from one study revealing that up to 12% of online gamers fulfilled diagnostic criteria for addiction [28]. However, as Charlton and Danforth [29] have pointed out, in terms of online gaming, of which MMORPG playing is but one manifestation, high engagement could easily be mistaken for addiction. However, as Ferguson et al. [30] advocate, the key issue is not to define a diagnostic category but to articulate whether or not particular online gaming behaviour actually or potentially interferes with the everyday activities of life or conversely augments the quality of intrapersonal and interpersonal functioning. In this review, a total of six studies were included [31–36] and were appraised using the McMaster University School of Rehabilitation Science quantitative [37] and qualitative [38] review forms.

### 2.3. Study Purpose

All of the reviewed studies examined the phenomenon of MMORPG playing in relation to the psychosocial well-being of adolescents and young adults. Justification for each study, including literature reviews, was provided however due to the exploratory nature of this area of research; each study had differing points of focus. Smyth [31] examined the effects of MMORPGs against other game types in terms of well-being, sleep, socialization, and academic work, whereas Frostling-Henningsson [32] sought to understand motivations for engaging in online gaming. Similarly, the studies by Kwon et al. [34] and Li et al. [36] aimed to explore any associations between escapism and online gaming. Lemola et al. [35] explored whether the amount and circadian time of online gaming was related to depressive symptoms, whilst Holtz and Appel [33] explored the link between online gaming and problem behaviour.

### 2.4. Study Design

Study designs have varying levels of rigour [39], and although only six studies were reviewed, when they are taken together a picture of both range and balance emerges. Five of the reviewed studies employed quantitative methodologies with the sixth utilising qualitative means. Four studies [33–36] used cross-sectional study designs and in terms of this review may be thought of as occupying the “middle ground” in the research methods continuum. Such designs provide data from a single moment in time from a particular cohort, and although they do provide some “answers” for the present [40], no causal inferences can be made. 

Alternatively, Smyth [31] conducted a prospective randomised trial in which participants were randomly assigned to different groups, thereby limiting the potential for bias or the influence of confounding variables [41]. In contrast, Frostling-Henningsson [32] used a mix of qualitative methods including observation, researcher introspection, and unstructured interviews and adopted a phenomenological approach. These remaining two studies can be, and often are, conceptualised as respectively inhabiting the levels above and below the other studies in the traditional hierarchy of evidence [42]. 

### 2.5. Ethics

Ethical approval is crucial in safeguarding the rights and interests of all concerned. Not only are researchers obliged to consider the wider impact of their research [43], but they should also indicate which particular measures were undertaken to ensure this is so [44]. All five quantitative studies described ethics procedures, such as approval from university ethics committees [31, 35] or school administrators, passive consent from parents, and assured participants of confidentiality [34, 36]. Holtz and Appel [33] indicate that participation in their study was voluntary, anonymous, and included informed consent. Only one study [32] omitted comment regarding ethics procedures. This may be due to the difficulties arising from the emergent nature of qualitative research in clearly outlining what the study will entail, and certainly the level of detail that is required to substantiate and situate qualitative research claims can compromise participant anonymity and confidentiality [45].

### 2.6. Sampling

Each study identified and recruited participants in a variety of ways; however, since the sampling strategy influences the external validity of the studies, the rigour and appropriateness of the methods used should be examined [45]. With one exception [31], all of the studies in this review utilised incidental sampling methods to access volunteer participants. This method is often used when a population is difficult to identify, the topic of study is controversial, or because of time and opportunity constraints. However, the weakness of this method is that there is no guarantee that it is a representative sample and as a consequence, the external validity of these results is weakened [46]. 

Three studies [33, 34, 36] accessed their participants through local secondary school populations, whilst all of the participants in the study by Smyth [31] were local university students, although it remains unclear how they were recruited. Lemola et al. [35] recruited participants through their responses to advertisements in online games and connected internet forums. In a somewhat similar fashion, Frostling-Henningsson [32] visited two different gaming centres in Stockholm and engaged participants face to face as the opportunities arose. On this occasion, however, the sampling technique is purposive and is entirely appropriate, given the aims and design of the study to explore participant experience of a particular phenomena. 

Sampling error remains a relevant question, with only two studies having samples of more than two hundred and fifty participants [34, 35]. Furthermore, error within the sample is of particular concern in the study by Lemola et al. [35], since all participant interaction took place online and there is no way to verify the self-reported demographic information provided. Moreover, the study by Kwon et al. [34] reported two hundred and sixty-four drop outs due to improperly checked questionnaires, which amounted to one-fifth of the original sample population.

### 2.7. Outcome Measures

Only two studies [34, 36] employed standardised outcome measures which had been validated for the research sample, providing high evidence of reliability with Cronbach's alpha scores ranging from 0.74–0.96 for each measure used. The strength of the psychometric properties of the measures adds significant weight to the results obtained. In contrast, the remaining quantitative studies [31, 33, 35] modified and adapted existing measures or developed their own to meet their particular research needs. In these instances, the data are not well supported by the rigour of standardised assessments, which are both reliable and valid for the particular population under study. Frostling-Henningsson [32], due to the qualitative nature of the study, tape recorded the interviews and transcribed two-thirds of these verbatim. None of the studies reported if those who administered the measures had the necessary training to do so [47].

### 2.8. Intervention

Of the six studies under review, only one incorporated active intervention [31], whereby one hundred participants (73 males and 27 females), all of whom were attending North American University, aged 18–20, were randomly allocated into four groups of twenty-five, with each group assigned to only play a specific genre of game for a period of one month, thereby isolating MMORPGs from other game types. The intervention required them to play their assigned game for a minimum of one hour per week at home. All necessary equipment was provided without cost to the participants, and whilst this allows for the study of the phenomena under experimental conditions for a finite period of time, it is questionable if one month is sufficient. The remaining studies did not incorporate any active intervention, choosing instead to gather information from participants in a single defined moment of time.

Holtz and Appel [33] administered their questionnaire during the summer to 205 pupils in Austrian schools (100 males and 105 females) aged 10–14 under the supervision of a research assistant and a class teacher. Kwon et al. [34] also conducted their questionnaire in schools to 1400 junior high pupils in Seoul, although no further details of administration are given. Again, Li et al. [36] administered questionnaires in school to 161 pupils in Singapore, with participants ranging from 13–15 years of age (49.1% male and 50.9% female). In contrast, Lemola et al. [35] administered their questionnaire online and report 646 responses from participants aged between 13 and 30 years old (91% male and 9% female). In a similar fashion, Frostling-Henningsson [32] conducted unstructured interviews and observations during the winter at two gaming centres in Stockholm with 23 gamers aged between 12 and 26 years old (19 males and 4 females). 

In all the studies, participants were familiar and comfortable with the study environment (home, school, gaming centre), and it may be argued that this could increase the veracity of the responses; yet for the participants completing questionnaires under the supervision of class teachers, in the presence of that existing power dynamic, arguments for the Hawthorne effect could be made [48]. Moreover, although quick information was generated in some cases, participants were required to exercise significant recall ability which can compromise the accuracy of the data. However, an interesting contrast emerged between two studies, one of which was conducted throughout the Scandinavian winter [32] with the other [33] taking place during an Alpine summer. Frostling-Henningsson [32] reported largely positive outcomes for study participants who played MMORPGs, whereas Holtz and Appel [33] identified mostly negative associations for the same phenomena. 

### 2.9. Data Analysis

Data analysis involves the application of statistical procedures to ultimately arrive at a unified statement concerning the research problem or question [49]. To that end, Holtz and Appel [33] initially used factor analysis to confirm a good model fit of three separate Internet elements, namely, information, communication, and gaming. Binary logistic regression was subsequently used, so that the independent variable (online gaming) could be used to predict the dichotomous dependent variable (internalising and externalising problem behaviour). On the other hand, Lemola et al. [35] used multiple regression analysis to explain the relationship between a number of independent variables and one dependent variable. These are regarded as comprehensive and appropriate statistical techniques. In a similar way, Kwon et al. [34] performed correlational and stepwise multiple regression analysis before conducting path analyses to determine if any statistically significant relationships existed amongst the variables. Li et al. [36] also correctly utilised path analysis to test relationships amongst variables, making comparisons between two models after using independent sample *t-*tests to differentiate between genders. 

Smyth [31] evaluated differences between experimental groups at one-month followup using analyses of variance and an omnibus *F*-test. According to Polgar and Thomas [46], this is an appropriate technique as the study was seeking to differentiate between four independent groups that were randomly assigned requiring a suitable parametric test. Frostling-Henningsson [32], however, used inductive and interpretive analysis to attempt to understand gamers' motivations. Since the author and the researcher are one and the same person, an independent secondary source of analysis would have strengthened the study. However, since not all of the interviews were transcribed and there is no indication that the researcher has identified and suspended their own personal views or preconceived ideas about the phenomenon [41], the chief concern would be that which Crombie [45] calls “serendipity masquerading as hypothesis.” Indeed, despite the attractiveness of the study, there is no way to be certain that the author is not merely constructing a picture using only selected data. 

## 3. Results and Discussion

### 3.1. Results

Kwon et al. [34] found that MMORPG playing became “pathological” for some adolescents and was significantly associated with three intrapersonal variables: real-ideal self-discrepancy, negative mood, and escape from self (all *P* < 0.001), with escape from self exerting the strongest influence, suggesting that engaging in the immersive worlds of MMORPGs was a method of alleviating negative and stressful thoughts within oneself. Furthermore, alongside that idea, their results indicate that pathological Internet gaming was more highly correlated with parental rather than peer relationships and was significantly associated with parental hostility and parental supervision (both *P* < 0.001) indicating that external stressors are also important. In a similar fashion, the study by Li et al. [36] also indicated that actual ideal self-discrepancy, escapism, and depression were significantly associated with “pathological” MMORPG playing (all *P* < 0.01), and when comparing statistics, their default model showed substantial data model fit which explained pathological online gaming as a function of all three factors (*P* < 0.05) indicating the complexity of the phenomenon and highlighting again the idea of MMORPG playing as a very modern coping strategy for individuals hoping to avoid negative emotions and to reduce discrepancies between their actual and ideal selves. For adolescents and emerging adults in particular, who remain engaged in the processes of self-exploration and development, this is a salient point.

Although Lemola et al. [35] did not find any evidence of effect of total playing time on depressive symptoms, they did find that there was a significantly increased risk of depression in thirteen to seventeen year olds habitually playing between 10 pm and midnight (*P* < 0.03) and eighteen to twenty-two year olds habitually playing between 2 am and 6 am (*P* < 0.04). Not surprisingly, daytime sleepiness was also significantly related to higher depressive symptom scores (*P* < 0.05). This study suggests that late night playing and night time playing are risk factors for negative health outcomes from MMORPG playing. Significantly, it could be the social and cooperative global nature and appeal of these games which may be demanding that players play at a time more “suitable” to other in-game colleagues in other parts of the world. Often in-game challenges must be completed by all team members, and it is worth considering whether a degree of cyber peer-pressure to be “awake and available” in order to complete team tasks is present.

Holtz and Appel [33] however identified a statistically significant association between online gaming and internalising problems, such as social withdrawal, anxiety, and depression (*P* < 0.05), which they did not find to be present for other Internet applications. Interestingly the social aspect of MMORPGs is often a key feature spoken of as bringing great enjoyment and benefit to game users; however, in this study it is postulated that by giving time to creating and developing on a new identity in-game, users are “distracted from real-world challenges and opportunities” and consequently withdraw from previous life roles and routines. This has often been called the “Internet paradox,” where communication technology may in fact lead to greater isolation and loneliness. Helpfully, the study by Smyth [31] revealed a number of statistically significant differences between groups of gamers. The MMORPG group reported playing more hours than control groups (*P* < 0.01), experiencing worse overall health (*P* < 0.05), poorer sleep quality (*P* < 0.05), and that game play had interfered with real-life socialising and academic work (both *P* < 0.05) to a greater extent. However, the study also found that MMORPG players experienced new online friendships to a greater degree (*P* < 0.01), enjoyed the playing experience more, and had a greater desire to continue playing (both *P* < 0.05). This study confirms that MMORPG games are “compelling, immersive, and socially rich environments” which can both cause difficulties and bring benefits to users. However, it would seem that the harmful psychosocial health effects outweigh the beneficial. Indeed, the question could be posed—are not the positive health experiences of MMORPG playing merely digital versions of that which is available in the real world? Emergent themes from the study by Frostling-Henningsson [32] indicate that sociality, cooperation, communication, control, and escapism are key motivating factors for young gamers. In this study, gamers are portrayed as individuals seeking comfort through controlled interaction with others in a place of refuge away from mundane trivialities and everyday problems, thus returning to the idea that MMORPG playing is a way to introduce into one's life those things which are deemed desirable but absent from real-world living and to avoid unpleasant and negative emotions and thinking. 

### 3.2. Discussion and Recommendations

The aim of this systematic review was to examine the literature and critically appraise the evidence to ascertain if playing MMORPGs influenced the psychosocial well-being of adolescents and young adults. In this regard, all six studies observed significant associations, and while no study is perfect [50], it is important to identify the positive contributions made to the evidence base by the reviewed research [51]. 

The study by Smyth [31] provides the strongest evidence for making causal statements about the effects of MMORPG playing, because the methodology employed counters the self-selection bias of individuals who would have elected to play this type of online game. In contrast, the study by Frostling-Henningsson [32] furnishes and enriches understanding through experientially situated data [52], while the cross-sectional studies [33–36] further broaden the horizons of this research area by exploring a slightly larger number of participants in a greater variety of contexts. Indeed in this regard, some have argued that a heterarchy rather than a hierarchy of evidence is more applicable in the face of such complexity [53, 54]. Furthermore, in contrast to the cross-sectional studies, it is interesting that the only study which looked at the phenomenon of MMORPG playing over time [31] found significant associations of positive as well as negative effect upon psychosocial well-being, leading some to question whether low psychosocial well-being is a cause or consequence of heavy gaming [55]. Indeed, others have begun to discuss and explore the potential of online gaming as a viable therapeutic modality [56, 57].

Where possible therefore, further clinical trials with pellucid methodologies, clear randomisation of participants, and robust experimental designs are needed in order to be able to articulate with greater confidence and integrity the nature and direction of these influences [58]. It is important, however, not to overlook the value of qualitative studies to enrich and deepen both researcher and clinician understanding [59]. Its highly contextualised and emergent nature [60] sits well within a client-centred and holistic philosophy which appreciates the full complexity of human occupation [61], including places, purpose, and meanings [62]. Furthermore, this type of investigation is ideally placed to expand the scope of the potential research area. For example, qualitative studies might examine the attitudes and experiences of parents or carers of adolescents who play MMORPGs, and the results could provide guidance concerning particular themes or factors which could be taken up by supplementary quantitative studies. 

Moreover, a greater specificity is required for each of the conceptual demographic groups. The studies in this review cover participants with an age range of twenty years which is not focused enough to fully appreciate the diverse and contrasting personal, social, and developmental needs, aims and motivations of these populations. Finally, longitudinal studies which take greater account of the environmental factors affecting the rhythms and routines of occupation, which can be particularly flexible and varied for these populations, would be desirable. For example, when contrasting the Frostling-Henningsson [32] and Holtz and Appel [33] studies, it is possible to identify the influence of both the physical and temporal frameworks within which the research was conducted, and familial, cultural, and socioeconomic contexts including attitudes, opportunity, and cost remain unexplored.

The clinical significance of these findings is considerable although clinicians would readily recognise that the studies do not convey the breadth and complexity of client need often encountered in community practice [41], where the accepted medical model of therapy may have little to do with the world outside the hospital or health centre [63]. To that end, therapists and clinicians are becoming increasingly aware that understanding both the meaning and process of such occupations is crucial to achieving collaboratively agreed manageable solutions [64]. 

## 4. Conclusion

The results of this systematic review strongly suggest that playing MMORPGs can impact upon the psychosocial well-being of adolescents and young adults; however, only tentative statements can be made about the nature of this impact. The findings present an incomplete picture but are indicative of a new research area exploring complex phenomena against a background of everprogressing technology. Certainly greater care needs to be taken to explicitly identify both the type of game and the gamer under study. Too often, studies have failed to make the distinctions necessary for meaningful comparisons and appear to have allowed the scope of the study to be dictated by the nature of convenience samples. Studies investigating whether or not MMORPG playing in particular and online gaming in general is a successful, persistent, or helpful method of alleviating stress from both within and without would be helpful. Indeed, following the “careers” of MMORPG gamers through this important developmental period and beyond could assist in profoundly clarifying the psychosocial impact of this genre of gaming. Overall, continued multidisciplinary research incorporating different professional foci, which employs both quantitative and qualitative methodologies, is recommended to further elucidate both the clinical implications and potentialities of this most modern of leisure occupations.

## References

[1] Caplan SE (2007). Relations among loneliness, social anxiety, and problematic internet use. *Cyberpsychology and Behavior*.

[2] Lam LT, Peng ZW (2010). Effect of pathological use of the internet on adolescent mental health: a prospective study. *Archives of Pediatrics and Adolescent Medicine*.

[3] Kormas G, Critselis E, Janikian M, Kafetzis D, Tsitsika A (2011). Risk factors and psychosocial characteristics of potential problematic and problematic internet use among adolescents: a cross-sectional study. *BMC Public Health*.

[4] Block JJ (2008). Issues for DSM-V: internet addiction. *The American Journal of Psychiatry*.

[5] Morgan C, Cotten SR (2003). The relationship between Internet activities and depressive symptoms in a sample of college freshmen. *Cyberpsychology and Behavior*.

[6] van Rooij AJ, Schoenmakers TM, van de Eijnden RJJM, van de Mheen D (2010). Compulsive internet use: the role of online gaming and other internet applications. *Journal of Adolescent Health*.

[7] Frangos CC, Frangos CC, Sotiropoulos I (2011). Problematic internet use among Greek university students: an ordinal logistic regression with risk factors of negative psychological beliefs, pornographic sites, and online games. *Cyberpsychology, Behavior, and Social Networking*.

[8] Blais JJ, Craig WM, Pepler D, Connolly J (2008). Adolescents online: the importance of internet activity choices to salient relationships. *Journal of Youth and Adolescence*.

[9] Morrison CM, Gore H (2010). The relationship between excessive internet use and depression: a questionnaire-based study of 1,319 young people and adults. *Psychopathology*.

[10] COMSCORE Worldwide online gaming community reaches 217 million people. http://www.comscore.com/Insights/Press_Releases/2007/07/Worldwide_Online_Gaming_Grows.

[11] Centres for Disease Control and Prevention eGames Data Brief. http://www.cdc.gov/healthcommunication/Research/DataBriefs/egamesresearch.pdf.

[12] NPD GROUP Video games experience significant growth in online gaming activities. http://www.npd.com/wps/portal/npd/us/news/press-releases/pr_090310a/.

[13] Byron T (2008). *Safer Children in A digital World: the Report of the Byron Review*.

[14] Wan CS, Chiou WB (2006). Why are adolescents addicted to online gaming? An interview study in Taiwan. *Cyberpsychology and Behavior*.

[15] Weaver JB, Mays D, Sargent Weaver S (2009). Health-risk correlates of video-game playing among adults. *American Journal of Preventive Medicine*.

[16] Porter G, Starcevic V, Berle D, Fenech P (2010). Recognizing problem video game use. *Australian and New Zealand Journal of Psychiatry*.

[17] Kim MG, Kim J (2010). Cross-validation of reliability, convergent and discriminant validity for the problematic online game use scale. *Computers in Human Behavior*.

[18] Kuss D, Griffiths MD (2012). Internet gaming addiction: a systematic review of empirical research. *International Journal of Mental Health and Addiction*.

[19] Allison SE, von Wahlde L, Shockley T, Gabbard GO (2006). The development of the self in the era of the internet and role-playing fantasy games. *The American Journal of Psychiatry*.

[20] Young K (2009). Understanding online gaming addiction and treatment issues for adolescents. *The American Journal of Family Therapy*.

[21] Pawlikowski M, Brand M (2011). Excessive Internet gaming and decision making: do excessive World of Warcraft players have problems in decision making under risky conditions?. *Psychiatry Research*.

[22] Messias E, Castro J, Saini A, Usman M, Peeples D (2011). Sadness, suicide, and their association with video game and internet overuse among teens: results from the Youth Risk Behavior Survey 2007 and 2009. *Suicide and Life-Threatening Behavior*.

[23] Shieh KF, Cheng MS (2007). An empirical study of experiential value and lifestyles and their effects on satisfaction in adolescents: an example using online gaming. *Adolescence*.

[24] Hussain Z, Griffiths MD (2009). The attitudes, feelings, and experiences of online gamers: a qualitative analysis. *Cyberpsychology and Behavior*.

[25] Longman H, O’Connor E, Obst P (2009). The effect of social support derived from world of warcraft on negative psychological symptoms. *Cyberpsychology and Behavior*.

[26] Demetrovics Z, Urbán R, Nagygyörgy K (2011). Why do you play? The development of the motives for online gaming questionnaire (MOGQ). *Behavior Research Methods*.

[27] Lent RW (2004). Toward a unifying theoretical and practical perspective on well-being and psychosocial adjustment. *Journal of Counseling Psychology*.

[28] Grüsser SM, Thalemann R, Griffiths MD (2007). Excessive computer game playing: evidence for addiction and aggression?. *Cyberpsychology and Behavior*.

[29] Charlton JP, Danforth IDW (2007). Distinguishing addiction and high engagement in the context of online game playing. *Computers in Human Behavior*.

[30] Ferguson CJ, Coulson M, Barnett J (2011). A meta-analysis of pathological gaming prevalence and comorbidity with mental health, academic and social problems. *Journal of Psychiatric Research*.

[31] Smyth JM (2007). Beyond self-selection in video game play: an experimental examination of the consequences of massively multiplayer online role-playing game play. *Cyberpsychology and Behavior*.

[32] Frostling-Henningsson M (2009). First-person shooter games as a way of connecting to people: ‘brothers in blood’. *Cyberpsychology and Behavior*.

[33] Holtz P, Appel M (2011). Internet use and video gaming predict problem behavior in early adolescence. *Journal of Adolescence*.

[34] Kwon JH, Chung CS, Lee J (2011). The effects of escape from self and interpersonal relationship on the pathological use of internet games. *Community Mental Health Journal*.

[35] Lemola S, Brand S, Vogler N, Perkinson-Gloor N, Allemand M, Grob A (2011). Habitual computer game playing at night is related to depressive symptoms. *Personality and Individual Differences*.

[36] Li D, Liau A, Khoo A (2011). Examining the influence of actual-ideal self-discrepancies, depression, and escapism, on pathological gaming among massively multiplayer online adolescent gamers. *Cyberpsychology, Behavior, and Social Networking*.

[37] Law M, Stewart D, Pollock N, Letts L, Bosch J, Wetmorland M 1998-last update. Guidelines for critical review form—quantitative studies [Homepage of McMaster University]. http://www.srs-mcmaster.ca/Portals/20/pdf/ebp/quanreview.pdf.

[38] Letts L, Wilkins S, Law M, Bosch J, Westmorland M 2007-last update. Guidelines for critical review form—qualitative studies (version 2, 0) [Homepage of McMaster University]. http://www.srs-mcmaster.ca/Portals/20/pdf/ebp/qualreview_version2.0.pdf.

[39] Macdermid J, Law M, Law M, Macdermid J (2008). Evaluating the evidence. *Evidence-Based Rehabilitation: A Guide to Practice*.

[40] Parahoo K (1997). *Nursing Research: Principles, Process and Issues*.

[41] Taylor MC (2007). *Evidence-Based Practice For Occupational Therapists*.

[42] Aveyard H (2010). *Doing a Literature Review in Health and Social Care: A Practical Guide*.

[43] Ingham-Broomfield R (2008). A nurses’ guide to the critical reading of research. *Australian Journal of Advanced Nursing*.

[44] Finlay L (1997). Evaluating research articles. *British Journal of Occupational Therapy*.

[45] Crombie IK (1996). *The Pocket Guide to Critical Appraisal*.

[46] Polgar S, Thomas SA (2008). *Introduction To Research in the Health Sciences*.

[47] Bowling A, Bowling A, Ebrahim S (2005). Techniques of questionnaire design. *Handbook of Health Research Methods: Investigation, Measurement and Analysis*.

[48] Berg KE, Latin RW (2008). *Essentials of Research Methods in Health, Physical Education, Exercise Science and Recreation*.

[49] Cooper H (1998). *Synthesizing Research: A Guide For literature Reviews*.

[50] Oliver D, Mahon SM (2006). Reading a research article part III: the data collection instrument. *Clinical Journal of Oncology Nursing*.

[51] Drummond A (1996). Reviewing a research article. *British Journal of Occupational Therapy*.

[52] Wilding C, Whiteford G (2005). Phenomenological research: an exploration of conceptual, theoretical, and practical issues. *OTJR Occupation, Participation and Health*.

[53] Tickle-Degnen L, Bedell G (2003). Heterarchy and hierarchy: a critical appraisal of the ‘levels of evidence’ as a tool for clinical decision making. *American Journal of Occupational Therapy*.

[54] White J, Crepeau EB, Cohn ES, Boyt Schell BA (2009). Questions for occupational therapy practice. *Willard and Spackman’s Occupational Therapy*.

[55] Lemmens JS, Valkenburg PM, Peter J (2011). Psychosocial causes and consequences of pathological gaming. *Computers in Human Behavior*.

[56] Freddolino PP, Blaschke CM (2008). Therapeutic applications of online gaming. *Journal of Technology in Human Services*.

[57] Shandley K, Austin D, Klein B, Kyrios M (2010). An evaluation of “Reach Out Central”: an online gaming program for supporting the mental health of young people. *Health Education Research*.

[58] Pope C, Mays N, Pope C, Mays N (2006). Observational methods. *Qualitative Research in Health Care*.

[59] Mays N, Pope C, Pope C, Mays N (2006). Quality in qualitative health research. *Qualitative Research in Health Care*.

[60] Bailey DM, Crepeau EB, Cohn ES, Boyt Schell BA (2003). Research: discovering knowledge through systematic investigation. *Willard and Spackman’a Occupational Therapy*.

[61] Creek J (2003). *Occupational Therapy Defined as a Complex Intervention*.

[62] Hasselkus BR (2011). *The Meaning of Everyday Occupation*.

[63] Pollard N, Kronenberg F, Creek J, Lougher L (2008). Working with people on the margins. *Occupational Therapy and Mental Health*.

[64] Daniel MA, Blair SEE, Duncan EAS (2011). An introduction to the psychodynamic frame of reference. *Foundations For Practice in Occupational Therapy*.

